# Evaluation of the dual mTOR/PI3K inhibitors Gedatolisib (PF-05212384) and PF-04691502 against ovarian cancer xenograft models

**DOI:** 10.1038/s41598-019-55096-9

**Published:** 2019-12-10

**Authors:** Simon P. Langdon, Charlene Kay, In Hwa Um, Michael Dodds, Morwenna Muir, Grant Sellar, Julie Kan, Charlie Gourley, David J. Harrison

**Affiliations:** 1 Cancer Research UK Edinburgh Centre and Edinburgh Pathology, Institute of Genetics and Molecular Medicine, University of Edinburgh, Edinburgh, EH4 2XU United Kingdom; 2 Nicola Murray Centre for Ovarian Cancer Research, Cancer Research UK Edinburgh Centre, Institute of Genetics and Molecular Medicine, University of Edinburgh, Edinburgh, EH4 2XU United Kingdom; 3 Pathology, School of Medicine, University of St. Andrews, North Haugh, St. Andrews, Fife KY16 9TF United Kingdom; 4 Wyeth Translational Medicine Research Consortium, Sir James Black Centre, Dow Street, Dundee, DD1 5EH United Kingdom; 5 Pfizer Translational Pharmacology, Oncology, San Diego, USA

**Keywords:** Cancer models, Ovarian cancer

## Abstract

This study investigated the antitumour effects of two dual mTOR/PI3K inhibitors, gedatolisib (WYE-129587/PKI-587/PF-05212384) and PF-04691502 against a panel of six human patient derived ovarian cancer xenograft models. Both dual mTOR/PI3K inhibitors demonstrated antitumour activity against all xenografts tested. The compounds produced tumour stasis during the treatment period and upon cessation of treatment, tumours re-grew. In several models, there was an initial rapid reduction of tumour volume over the first week of treatment before tumour stasis. No toxicity was observed during treatment. Biomarker studies were conducted in two xenograft models; phospho-S6 (Ser235/236) expression (as a readout of mTOR activity) was reduced over the treatment period in the responding xenograft but expression increased to control (no treatment) levels on cessation of treatment. Phospho-AKT (Ser473) expression (as a readout of PI3K) was inhibited by both drugs but less markedly so than phospho-S6 expression. Initial tumour volume reduction on treatment and regrowth rate after treatment cessation was associated with phospho-S6/total S6 expression ratio. Both drugs produced apoptosis but minimally influenced markers of proliferation (Ki67, phospho-histone H3). These results indicate that mTOR/PI3K inhibition can produce broad spectrum tumour growth stasis in ovarian cancer xenograft models during continuous chronic treatment and this is associated with apoptosis.

## Introduction

Worldwide, there are approximately 239,000 new cases of ovarian cancer each year accounting for 4% of all cancers diagnosed in women^1^. Standard therapy consists of maximal surgical debulking and 6 cycles of platinum-taxane combination chemotherapy. Although most ovarian cancers are initially chemosensitive, the majority relapse and ultimately become chemoresistant^2,3^. Novel therapies are required and since components of the phosphoinositide-3-kinase (PI3K) pathway are frequently modified in ovarian cancer, targeting this pathway may be beneficial in this disease^4–6^.

PI3K activates mTOR via AKT and the pathway is activated in 70% of ovarian cancers^3^. Elevated phospho-Akt (Ser473) is found in 68% of ovarian cancers and phospho-mTOR in 55% of tumours^7^. Constitutive activation and deregulation of the PI3K/AKT/mTOR pathway may involve mutational activation or amplification of PI3K family members, loss of PTEN function, amplification of the AKT isoenzymes AKT1 and AKT2 or inactivation or mutation of AKT-associated mTOR-regulating molecules TSC1 or TSC2^4–8^. In high grade serous ovarian cancer (HGSOC), the predominant type of ovarian cancer, it is estimated that the PI3K/AKT/mTOR pathway is activated in about half of cancers, with frequent amplifications in PIK3CA, AKT1, AKT2 and deletions in PTEN while mutations in the pathways components are relatively uncommon (<5% cancers)^4–6,8^. By contrast, in non-HGSOC e.g. clear cell or endometrioid subtypes, mutational activation is much more frequent and present in up to 30% of cancers^4–6^.

Multiple inhibitors of the PI3K pathway have been developed and are under consideration for use in ovarian cancer^4–6,9^. They include PI3K/AKT inhibitors, mTOR inhibitors, and dual PI3K/mTOR inhibitors. This last group were developed after demonstration that due to the existence of an S6K1-based negative feedback loop, prolonged mTOR inhibition leads to enhanced PI3K/AKT activation^10–13^. This could be overcome by use of inhibitors that simultaneously target both PI3K and mTOR. Two such inhibitors are PF-04691502^14,15^ and gedatolisib (also known as PF-05212384, WYE-129587or PKI-587)^16^. PF-04691502 has shown interesting activity against a large panel of ovarian cancer cell lines *in vitro*^17^. Both compounds have now entered Phase I clinical trials^18,19^.

The purpose of this study was to assess the antitumour efficacy of these two dual mTOR/PI3K inhibitors in patient derived xenograft models of ovarian cancer. Use of a panel of xenografts allowed assessment of the variation of any effects and characterization of the linkage between antitumour response and signalling. Markers of cell signalling and function were measured to provide support that the pathway was being inhibited and to provide information on the functional consequences.

## Results

### Assessment of the antitumour activity of Gedatolisib and PF-04691502 against a panel of xenograft models

A panel of six human ovarian cancer xenografts (OV1002, HOX 424, HOX 516, HOX 552, HOX 299, HOX 493) were used in this study and four of these (HOX424, OV1002, HOX 516, HOX 493) have been described previously^20–22^. OV1002, HOX 516 and HOX 552 were derived from HGSOC, HOX 424 was derived from an endometrioid ovarian cancer while HOX 552 and HOX493 were of clear cell origin.

**Figure 1:**
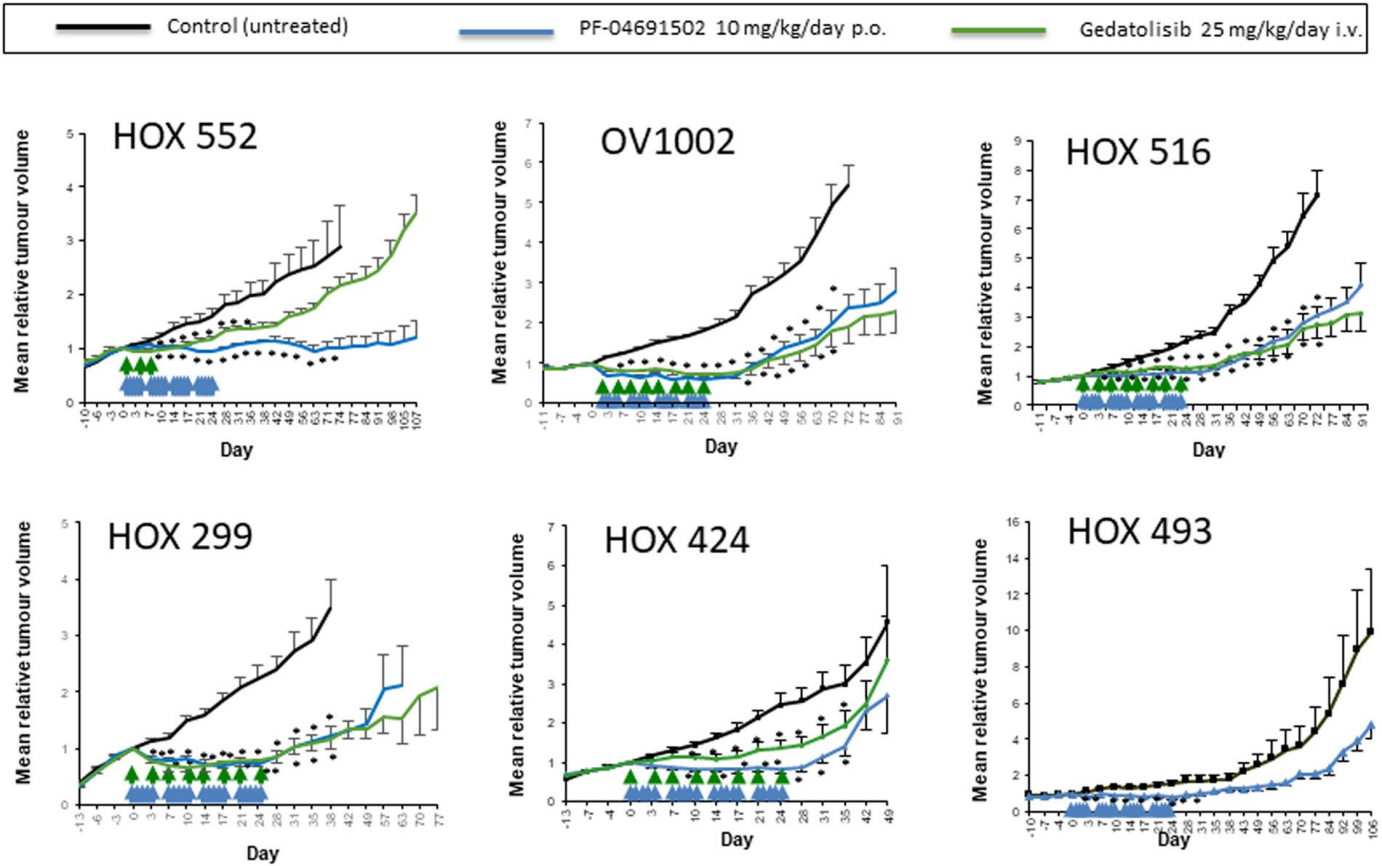
Antitumour activity of PF-04691502 and gedatolisib against a panel of human ovarian cancer xenografts. Both drugs were evaluated against the HOX 552, OV1002, HOX 516, HOX 299, HOX 424 models. PF-04691502 only was tested against the HOX 493 model. PF-04691502 (10 mg/kg/day p.o.) and gedatolisib (25 mg/kg/day i.v.) were administered on the days indicated by arrows. PF-04691502 was administered over 4 weeks (days 0–4; 7–11; 14–18; 21–25) against all 6 models while gedatolisib (25 mg/kg) was given on days 0, 4 and 8 only to HOX 552 but then extended to days 0, 4, 7, 11, 14, 18, 21 and 25 for OV1002, HOX516, HOX299, HOX424. Mean (+/− S.E.) tumour volumes are shown. For OV1002 and HOX 299, all treatment points shown were statistically different from control (ANOVA followed by Tukey post-test; *p < 0.05). For HOX 516, all treatment points beyond Day 3 were statistically different from control. For HOX 552, all treatment points were statistically different from control beyond Day 10 until Day 36 for gedatolisib and Day 71 for PF-04691502. For HOX424, all treatment points after Day 7 and before Day 35 were statistically different from control. For HOX 493, all treatment points were statistically different from control until Day 31.

The two mTOR/PI3K inhibitors, Gedatolisib (1-[4-[4-(dimethylamino)piperidine-1-carbonyl]phenyl]-3-[4-(4,6-dimorpholin-4-yl-1,3,5-triazin-2-yl)phenyl]urea) and PF-04691502 (2-Amino-8-[trans-4-(2-hydroxyethoxy) cyclohexyl]-6-(6-methoxypyridin-3-yl)-4-methylpyrido[2,3-d] pyrimidin-7(8 H)-one), were first assessed against the HOX 552 ovarian cancer xenograft model. PF-04691502 (10 mg/kg/day) was administered p.o. over 4 weeks (days 0–4; 7–11; 14–18; 21–25) while gedatolisib (25 mg/kg) was given i.v. on days 0, 4 and 8 only. Both drugs produced a growth delay during the treatment period with PF-04691502 producing a longer lasting effect than gedatolisib (Fig. 1). On discontinuation of either drug, the treated tumours regrew.

A further four xenografts (OV1002, HOX516, HOX299, HOX424) were then tested using the same schedule for PF-04691502 but extending the schedule for gedatolisib (days 0, 4, 7, 11, 14, 18, 21 and 25) (Fig. 1). A sixth xenograft, HOX 493, was treated with PF-04691502 only (Fig. 1). Using this extended schedule for gedatolisib resulted in activity comparable to that of PF-04691502 in the OV1002, HOX516 and HOX299 xenograft models. For OV1002 and HOX299, there was an initial decrease in tumour volume during the first 3 days of treatment with PF-04691502 which then stabilized over the treatment period. This initial decrease was less obvious for gedatolisib. On cessation of treatment, tumours regrew. For the HOX 516 xenograft there was no evidence of an initial decrease in tumour volume but significant tumour stasis was achieved while treatment was given. For the HOX 424 xenograft, both drugs had significant activity against this model, however the gedatolisib-treated tumours showed some increase in growth during drug treatment which contrasted with the other models tested. There was no significant change in mean body weight of the animals during drug treatment (Supplementary Fig. 1) or any other indication of toxicity in these experiments.

In summary, both PF-04691502 and gedatolisib were active against all models tested. A broadly similar response was achieved in all cases with consistent static inhibition of tumour growth across all models and reduced activity of gedatolisib against the HOX 424 model. In some models, there was evidence of an initial reduction in growth over the first 3–7 days but in all cases, this converted to a static effect.

### Effects of Gedatolisib and PF-04691502 on PI3K/mTOR signalling in the xenograft models

Two xenograft models were then used to study pharmacodynamic changes within tumours. The OV1002 model was selected as it had shown marked sensitivity to both dual inhibitors while HOX424 showed reduced sensitivity to gedatolisib hence might demonstrate differential effects. Tumours were treated with the same schedule as used previously with the addition of two further oral injections for PF-04691502 on Days 28 and 29 and an extra i.v. injection for gedatolisib on Day 29. The tumour volume changes on treatment for this experiment are shown in Supplementary Fig. 2. Tumours were collected 4 h after injection on days 4, 7 and 29. Tumours were also collected on Day 46, 17 days after the last injection when tumours were regrowing. Untreated control tumours were collected on days 0 and 29. As with the antitumour studies, there was no change in mean body weight of the treated animals (Supplementary Fig. 3).

To analyse protein expression of selected markers within these xenografts after treatment, two related approaches were used. Tissue microarray (TMA) blocks of the xenografts were prepared and sections from these blocks assessed by standard immunohistochemistry (IHC) and by quantitative immunofluorescence (IF) using AQUA (automated quantitative analysis) methodologies^23^. TMA sections were cut and stained with a number of antibodies. These included phospho-S6 (pS6) and total S6 to evaluate mTOR inhibition and phospho-AKT (pAKT) and total AKT to evaluate PI3K inhibition. Markers of function including Ki67 (proliferation, cycling cells), phospho-histone H3 (mitotic cells) and apoptosis (apoptotic bodies) were also assessed.

**Figure 2:**
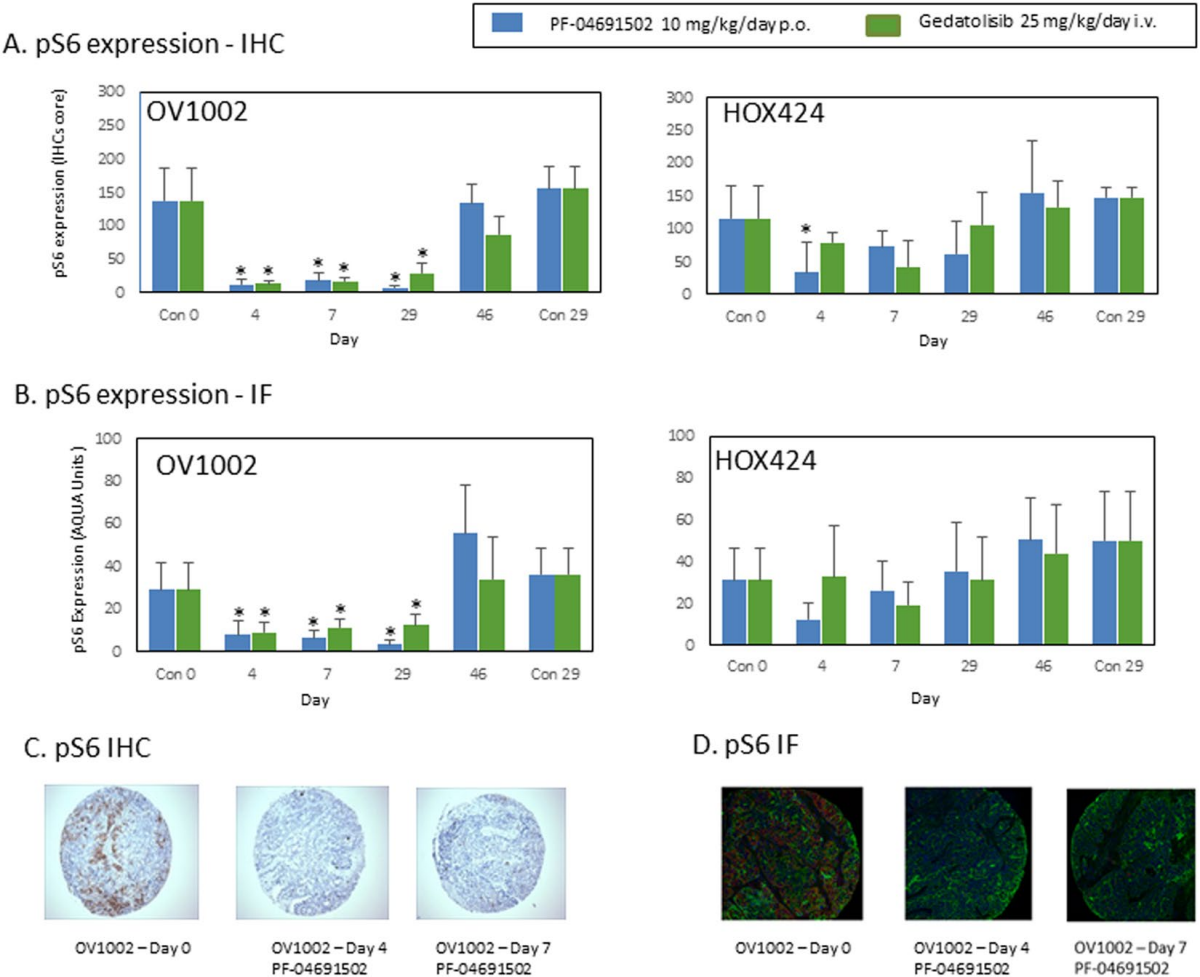
Expression of pS6 in OV1002 and HOX424 xenografts after treatment with PF-04691502 and gedatolisib. (**A**) pS6 (Ser235/236) expression detected by immunohistochemistry. Immunoscores are shown. (**B**) pS6 (Ser235/236) expression detected by immunofluorescence. Normalised AQUA scores are shown. (**C**) Examples of pS6 (Ser235/236) immunostaining in TMA cores. (**D**) Examples of pS6 (Ser235/236) immunofluorescence staining in TMA cores. Mean values shown are average values +/− S.D. for each group of xenografts. *P < 0.05 shown for ANOVA followed by Tukey post-test.

Treatment with either PF-04691502 or gedatolisib markedly reduced pS6 expression in OV1002 xenografts as assessed by either IHC or quantitative IF (Fig. 2). Illustrative examples are shown in Supplementary Figs. 4 and 5 for each day. The mean values for pS6 expression in OV1002 as detected by IHC or IF after treatment are depicted in Fig. 2A,B respectively. Phospho-S6 (pS6) expression was significantly reduced throughout the treatment period and returns to control values on discontinuation of treatment. Total S6 expression values as measured by AQUA did not decrease significantly relative to Day 0 control values (Supplementary Fig. 6).

For HOX 424 xenografts, the effect of PF-04691502 on HOX 424 as detected by IHC is shown in Fig. 2A. PF-04691502 produced a significant reduction in pS6 expression on Day 4 while gedatosilib produced a non-significant reduction on Day 7 (Fig. 2A). When pS6 expression was detected by AQUA analysis, it was reduced by PF-04691502 on Day 4 period and on Day 7 for gedatolisib, however these results did not achieve significance (Fig. 2B). With both agents, even on treatment, the levels of pS6 expression appeared to increase after an initial drop. Total S6 expression values as measured by AQUA again didn’t significantly decrease relative to Day 0 control values (Supplementary Fig. 6).

**Figure 3:**
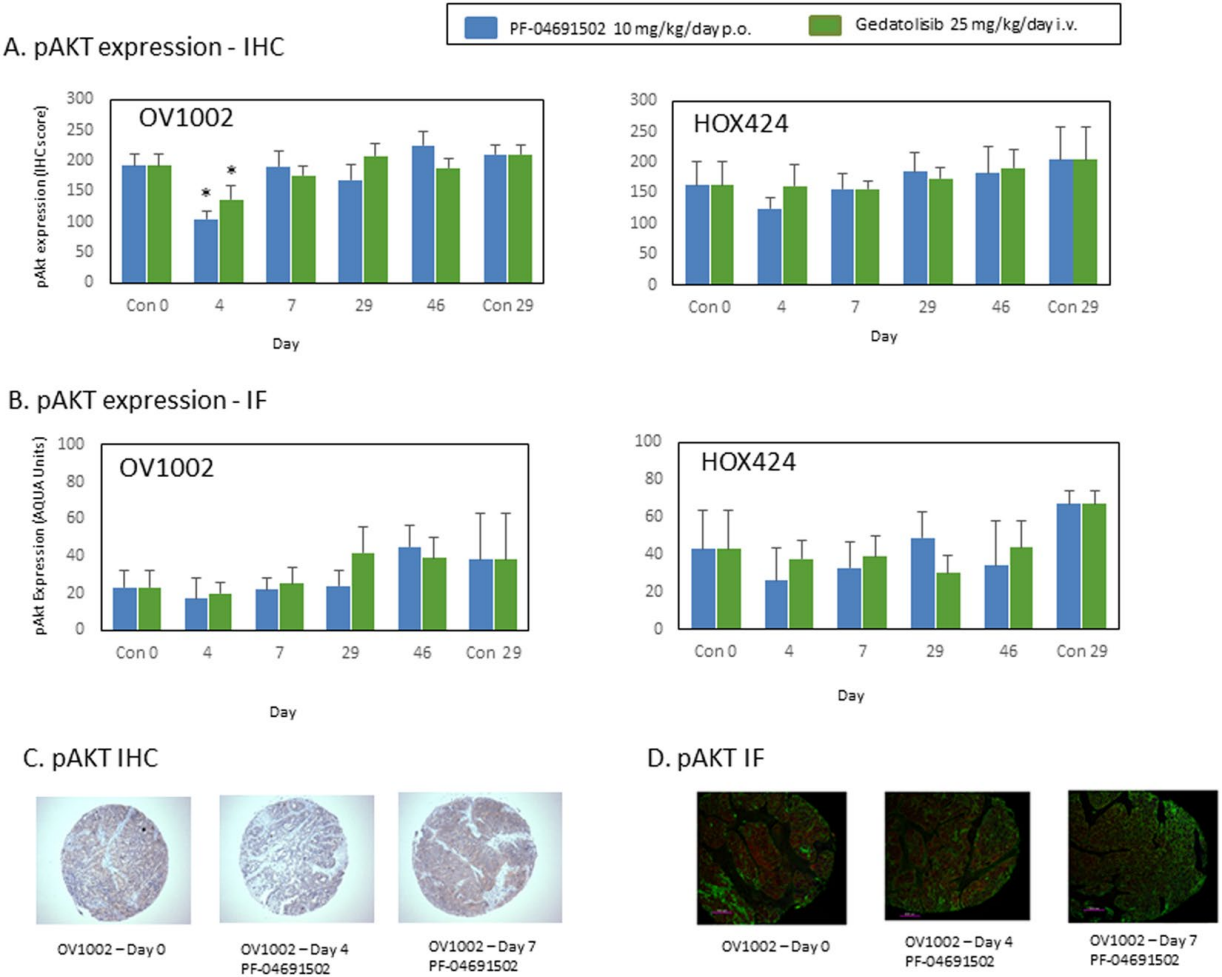
Expression of pAKT (Ser473) in OV1002 and HOX424 xenografts after treatment with PF-04691502 and gedatolisib. (**A**) pAKT (Ser473) expression detected by immunohistochemistry. Immunoscores are shown. (**B**) pAKT (Ser473) expression detected by immunofluorescence. Normalised AQUA scores are shown. (**C**) Examples of pAKT (Ser473) immunostaining in TMA cores. (**D**) Examples of pAKT (Ser473) immunofluorescence staining in TMA cores. Mean values shown are average values +/− S.D. for each group of xenografts. *P < 0.05 shown for ANOVA followed by Tukey post-test.

The expression of pAKT was more homogeneous than that for pS6 in the OV1002 xenograft model (Fig. 3A,B). Illustrative examples after treatment are shown in Fig. 3C,D. Significant reductions in pAKT expression were observed for PF-04691502 and gedatolisib on Day 4 when assessed by IHC (Fig. 3A). However, this reduction was not significant when measured by AQUA in TMA cores (Fig. 3B). Although significant changes were not observed in pAKT expression using triplicate cores, an analysis of large numbers of fields (10–30) was undertaken for each xenograft section and significant changes were observed in OV1002 for both PF-04691502 (on days 4 and 7) and gedatolisib on Day 7 (p < 0.05; ANOVA). This analysis did suggest that changes in expression were occurring but the effects were small and not easily detected. While pAKT was reduced on selected days, total Akt expression was not reduced on this analysis (Supplementary Fig. 6).

### Effects of Gedatolisib and PF-04691502 on apoptosis and proliferation markers within the xenograft models

**Figure 4:**
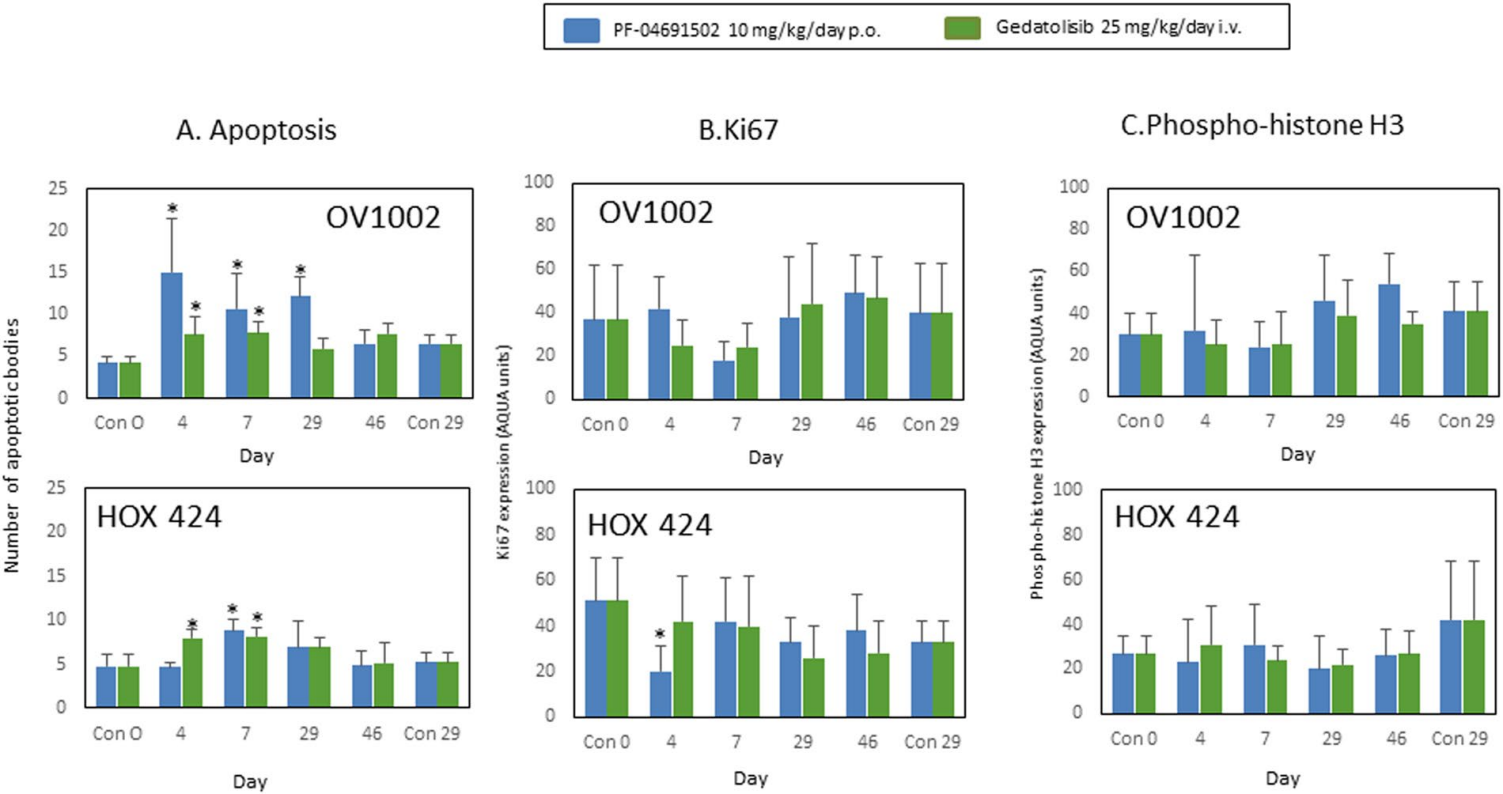
Apoptosis, Ki67 and phospho-histone H3 expression in OV1002 and HOX 424 xenografts after treatment with PF-04691502 and gedatolisib. (**A**) Mean number of apoptotic bodies in a high-field (x 40) view for OV1002 xenografts and HOX 424 xenografts. Mean values shown are average values for each group of xenografts. Ten fields were evaluated for each individual xenograft and the mean value was evaluated. (**B**) Ki67 expression. Mean % Ki67 expression is shown. C. Phospho-histone H3 expression. Mean % value (+/− S.D.) is shown for each group. *P < 0.05 shown for ANOVA followed by Tukey post-test.

Next, the effects of the drugs on indicators of functional change were investigated. To assess whether the two agents influenced apoptosis, the number of apoptotic bodies were counted in H&E sections using microscopy. Ten high powered fields (x 40) were evaluated for each xenograft and the mean value/single field was calculated. The mean of the group+/− S.D. is shown in Fig. 4A. Both drugs produced increases in apoptosis in both xenograft models at early time points most notably on days 4 and 7.

Two indicators of proliferation were next measured – Ki67 and phospho-histone H3. Ki67 is expressed during G_1_, S, and G_2_ phases of cell cycle with a peak during mitosis and an absence in G_0_ phase^24^. Phospho-histone H3 (Ser10) is expressed during mitosis^25^. Ki67 expression did not change significantly after treatment with either drug in OV1002 xenografts (Fig. 4B) while PF-04691502 had a small effect in the HOX424 xenograft, reducing Ki67 on day 4 only (Fig. 4B). Phospho-histone H3 (Ser10) was unchanged after treatment in xenografts (Fig. 4C).

These results suggest that induction of apoptosis rather than inhibition of proliferation is predominantly responsible for the initial tumour volume reduction and stasis.

### Association of Gedatolisib and PF-04691502 on pS6/S6 and pAKT/AKT ratios across the xenograft models

**Figure 5:**
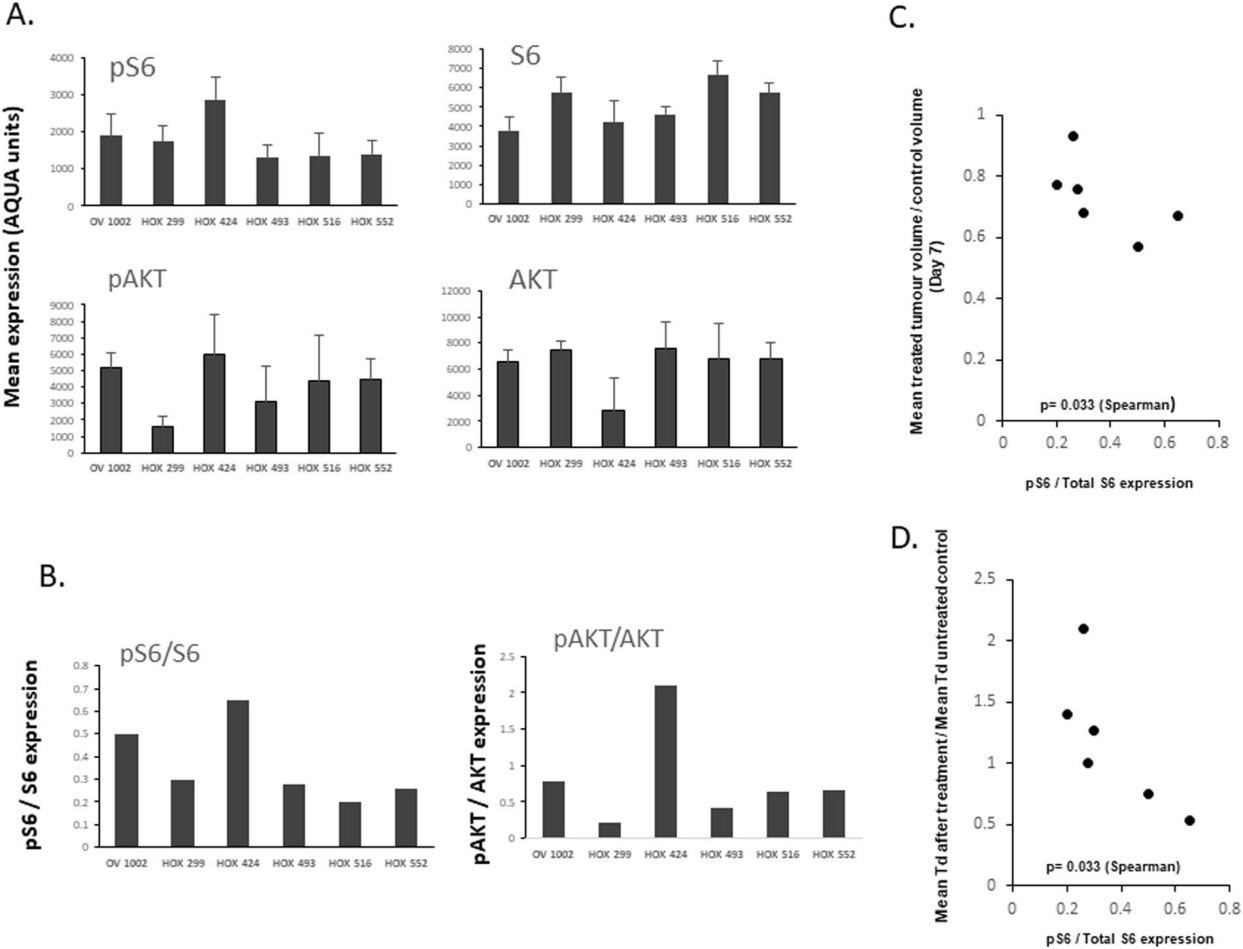
Association between pS6/total S6 expression and tumour volume index in 6 human ovarian cancer xenografts. (**A**) Baseline pS6, S6, pAKT and AKT mean values are shown for each xenograft model. (**B**) Ratios of pS6/S6 and pAKT/AKT are shown for each model. (**C**) Association between pS6/S6 expression plotted against tumour volume change after PF-04691502 treatment over 7 days. (**D**) Association between pS6/S6 expression plotted against the ratio of mean tumour volume doubling time (Td) at cessation of PF-04691502 treatment and mean tumour volume doubling time of untreated control.

We then analysed pS6 and pAKT to assess whether the level of expression of phospho-activated molecules might be associated with size of response to drug. Since this comparison was now between different xenograft models as opposed to the time course studies which are within individual models, phospho-activated expression was compared to the level of total expression (Fig. 5A,B). An association (p = 0.033 (Spearman)) was observed (Fig. 5C) between pS6/total S6 expression and tumour volume index (Day 7) after treatment with PF-04691502 suggesting that a higher level of initial pS6 expression was associated with a greater initial response on treatment.

Analysis of the growth rate of tumours after cessation of treatment also indicated interesting differences in the panel of xenografts. The mean doubling times of xenografts after the cessation of treatment with PF-04691502 was analysed (from Day 28 onwards) and compared with the initial mean doubling time of the control group (from Day 0) (Supplementary Table 1). These ratios (Supplementary Table 1) were then compared with the ps6/total S6 expression ratios and a significant association was again observed (p = 0.033 (Spearman)) (Fig. 5D). These results suggest that a higher ratio of pS6/total S6 expression is indicative of an initially enhanced response to PF-04691502 but then on discontinuation of drug, the tumours relapse more rapidly.

## Discussion

The data obtained with the two dual mTOR/PI3K inhibitors indicated broad spectrum activity against these preclinical models of ovarian cancer. The drugs produced a predominantly static effect against the xenograft models though in some cases there was an initial tumour volume reduction. In most models, the inhibitory effects ceased relatively quickly on cessation of drug treatment. There was no obvious toxicity as indicated by little or no body weight loss (Supplementary Fig. 1). The similarities in activity between the two drugs is consistent with both drugs having the same mechanism of action. Further work is needed to assess how long stable disease can be maintained on continuous drug treatment or whether resistance will emerge.

The biomarker studies completed to date suggest possible utility of pS6 as an indicator of response in tumour biopsies. A previous *in vitro* study evaluating PF-04691502 against 33 ovarian cancer cell lines *in vitro* concluded that sensitivity to the drug did not correlate with PI3 kinase activating mutations or PTEN loss in this disease^17^. Our analysis suggests early inhibition of mTOR signalling as indicated by reduction of pS6 expression but perhaps only short term control of pAKT expression. The inhibition of both pathways leads to an early decrease in tumour volume in some models but this appears to convert to stasis if only mTOR inhibition is sustained. Increased apoptosis rather than reduced proliferation was associated with growth stasis. Previous *in vitro* studies using ovarian cancer cell lines have associated mTOR inhibition with increased apoptosis^26^. In breast cancer cell line models, greater growth inhibition was observed in luminal and HER2-positive cell lines than in triple-negative cell lines after treatment with PF-04691502, with apoptosis being observed in the former^27^.

The initial tumour volume inhibition appeared to be strongest where pS6/total S6 expression was highest suggesting that tumours with the greatest pathway activation might be inhibited more. Similarly, on cessation of treatment, the xenografts regrew most rapidly where this ratio was greatest again supporting dependency on this pathway. This data supports the possible use of the pS6/total S6 ratio as a potential biomarker to help predict response but also potential rapid regrowth if the drug is stopped; this will require further validation.

On the basis of these results, these dual inhibitors have broad spectrum activity against ovarian cancer models producing disease stabilization rather than disease response (although some models did show an initial partial response). This could be valuable in extending time to progression when used as a maintenance therapy following chemotherapy. Both HGSOC and non-HGSOC xenografts responded to this treatment, which is not unexpected as these pathways are activated in multiple histological subtypes of ovarian cancer. The inhibitors were active when given continuously and cessation of treatment led to tumour regrowth. In these *in vivo* models in mice, disease stabilization was produced at doses that did not appear to have obvious toxicity.

These xenograft models may be of value in helping to define further the dynamic changes occurring on treatment. It is possible that feedback mechanisms are limiting the pAKT inhibition and further studies might identify these pathways within this xenograft material. If this were the case, use of other targeted inhibitors in conjunction with the mTOR/PI3K inhibitors might produce more potent antitumour effects.

Both PF-04691502 and Gedatolisib (PF-05212384) have now progressed through Phase I clinical trials^18,19^ and are being studied in combination with chemotherapy and inhibitors of other targeted pathways^28^. Within the phase I trial for PF-04691502, no responses were reported but stable disease was observed in 33% of patients^18^. Fatigue and rash were dose-limiting. A partial blockade of p-AKT (Ser473) signalling was observed in a group of 5 pre- and post-treatment biopsies^18^. In the Phase I trial of Gedatolisib, two partial responses (2.6%) were observed and disease stabilisation in 35% of patients. Comparison of tumour biopsies indicated a mean 30% reduction if p-AKT (Ser473) in a series of 8 pre-/post-treatment biopsies^19^. Our results indicate that it would be worthwhile evaluating changes in pS6 expression also in future studies with these drugs.

A multi-arm Phase I study which investigated both drugs in combination with either irinotecan or the MEK inhibitor PD-0325901 reported preliminary evidence of activity of gedatolisib plus the MEK inhibitor PD-0325901 in 3 ovarian cancer patients (all partial responses) out of 5 tested^28^. In cell line xenograft models (SKOV3 and A2780) of ovarian cancer, gedatolisib has shown enhanced antitumour activity when combined with crizotinib (a c-Met inhibitor) although its single agent activity was very limited^29^.

In conclusion, these results support continued consideration of these inhibitors for use in ovarian cancer.

## Methods

### Xenograft studies

The xenografts had been previously established in adult female CD-1 nude mice (housed in sterile isolators) directly from clinical material (ascites) and then passaged a minimum number of times until growth was reproducible and sufficient material was available to undertake experiments (4–10 passages). For the experiments described, groups of mice were implanted with fragments, subcutaneously in each flank. Group size was a minimum of 6 mice/group with at least 8 tumours/group. Tumours were allowed to grow to a group mean size of greater than 100 mm^3^ before drug treatment was initiated. PF-04691502 (2-Amino-8-[trans-4-(2-hydroxyethoxy) cyclohexyl]-6-(6-methoxypyridin-3-yl)-4-methylpyrido [2,3-d] pyrimidin-7(8 H)-one was prepared as a suspension in 0.5% carboxymethylcellulose (CMC) in sterile distilled water. Gedatolisib (1-[4-[4-(dimethylamino)piperidine-1-carbonyl]phenyl]-3-[4-(4,6-dimorpholin-4-yl-1,3,5-triazin-2-yl)phenyl]urea) was prepared as a suspension in 4% ethanol, 2% Tween and 4% PEG 400 in distilled water. In the initial experiment using HOX552, PF-04691502 (10 mg/kg/day) was administered by oral gavage over 4 weeks (days 0–4; 7–11; 14–18; 21–25) while gedatolisib (25 mg/kg/day) was given intravenously in the tail vein on days 0, 4 and 8 only. In the subsequent experiments, gedatolisib was administered on days 0, 4, 7, 11, 14, 18, 21 and 25. Tumour volumes were measured on the days shown in the figures using vernier calipers and calculated as volume = π/6 × l × w^2^ where l is the length of the tumour and w = width. In a separate experiment, a time course study over the drug treatment period was undertaken and tumours collected at the time points indicated. Tumours were collected 4 h after injection on the day described. Samples were placed into formalin as fixative and were subsequently embedded (FFPE) into paraffin wax. The xenograft studies were undertaken under a UK Home Office Project Licence in accordance with the Animals (Scientific Procedures) Act 1986 and studies were approved by the University of Edinburgh Animal Ethics Committee.

### Tissue microarray preparation and immunohistochemistry studies

For analysis of protein expression, three replicate tissue microarrays (TMAs) were prepared which used triplicate cores from each of the xenografts in the study. Sections were then cut from these TMA blocks.

A standard Envision immunohistochemistry protocol was used to assess proteins of interest^23^. Briefly, sections were de-waxed in xylene followed by rehydration. Antigen retrieval was undertaken in a sodium citrate buffer pH 6.0 for 10 min. Slides were placed into distilled water, then washed in PBST twice for 5 min. Sections were placed into 3% H_2_O_2_ for 10 min, then washed 3 times with PBST. Sections were added to Sequenza and washed with PBST. These were blocked with DAKO total protein block for 10 min. Primary antibody was diluted with DAKO antibody diluent and sections incubated in primary antibody either for an hour at room temperature or overnight at 4 °C. The following rabbit primary antibodies were used at the stated dilution; pS6 (Ser235/236) (Cell Signalling #2211) (1 in 200); S6 (Cell Signalling #2217) (1 in 100); pAKT (Ser473) (Cell Signalling #4060) (1 in 100) and AKT (pan) (Cell Signalling #4685) (1 in 200). Sections were washed 3 times with PBST, followed by incubation with DAKO Envision labeled polymer for 30 min at room temperature. Slides were washed 3 times with PBST. DAB (DAB substrate: DAB chromagen = 1 ml: 20ul) was added to visualize staining. Sections were washed in water and counterstained in hematoxylin (15–20 sec) followed by Scot’s tap water for 15–20 sec. Slides were dehydrated 2 min: 50%, 80%, 99%, 99% alcohol and then mounted. Expression was measured by a scoring system consisting of the product of the percentage of positively stained tumour cells and intensity of staining (0–3) producing an immunoscore ranging from 0 to 300. For assessment of apoptotic counts, H&E sections were assessed. Ten high field (x 40) areas were counted and the mean value calculated. Apoptotic bodies were identified as membrane enclosed bodies with condensed/fragmented nuclei and frequently with eosinophilic cytoplasm.

### Quantitative immunofluorescence (AQUA) studies

For assessment of protein expression by quantitative immunofluorescence, the following protocol was used^23^. Briefly, TMA sections were de-waxed in xylene 2 × 5 min. This was followed by rehydration for 2 min in 99%, 99%, 80% and 50% alcohol. Antigen retrieval was undertaken in a sodium citrate buffer pH 6.0 where slides were boiled for 5 min. They were washed in PBST (PBS/Tween) twice for 5 min. Sections were then placed in 3% H_2_O_2_ for 10 min, then washed 3 times with PBST. Sections were added to sequenza and washed with PBST. These were blocked with DAKO total protein block for 10 min. For mouse primary antibody, this was diluted at optimal dilution with second primary antibody (rabbit anti-cytokeratin, DAKO, Z0622, 1:150 dilution) diluted in DAKO antibody diluent either for an hour at room temperature or overnight at 4 °C. The same primary antibodies for pS6, S6, pAKT and AKT as used for IHC were used for immunofluorescence at the same dilutions while phospho-histone H3 (Ser10) (Cell Signalling #9701S) was used at 1 in 100 dilution and anti- Ki67 (DAKO M7240) was used at a 1 in 50 dilution. For rabbit primary antibody, the same protocol was used except the second primary antibody used was mouse anti-cytokeratin, Dako, M3515 diluted in Dako antibody diluent with 1:50 dilution overnight at 4 °C. Epithelial mask visualization then required a 1:25 dilution of the goat anti-mouse Alexa555 Ab (Invitrogen, #A21422) in the pre-diluted Envision goat-rabbit HRP antibody solution (Dako, #K4003). Sections were washed 3 times with PBST. A 1:25 dilution of the goat anti-rabbit Alexa555 Ab (Invitrogen, #A21428) in the pre-diluted Envision goat-mouse HRP antibody solution (Dako, #K4001) was prepared and added to slides which were then rinsed in 0.05% PBST 3 × 5 min. Slides were transferred from Sequenza to the humidity chamber. For target visualization, target signal amplification diluent and the Cy5 Tyramide at 1:50 concentration were combined. These were vortexed to mix thoroughly. Slides were incubated with solution in the dark for 10 min at room temperature. Slides were washed 3 times with PBST. These were then dehydrated in 80% IMS for 1 min and air dried in the dark. For counterstaining and coverslipping, 45ul Prolong Gold anti-fade reagent with DAPI (Invitrogen, P36931), nuclear visualisation media, was applied on the coverslip (22 × 40 mm) and the coverslip was placed over the tissue. The mounted slide was left to dry overnight in the dark. After slides were completely dried, the coverslips were sealed. Monochromatic images of each TMA core were captured at x20 objective using an Olympus AX-51 epifluorescence microscope, and high-resolution digital images analysed by the AQUAnalysis^TM^ software^23^.

## Supplementary information

Supplementary information
