# Supplementary material for: Evaluation of the dual mTOR/PI3K inhibitors Gedatolisib (PF-05212384) and PF-04691502 against ovarian cancer xenograft models
Source: Sci Rep. 2019 Dec 10;9:18742. doi: 10.1038/s41598-019-55096-9 (PMC6904563; doi:10.1038/s41598-019-55096-9)
Supplement: Supplementary file 1 — Supplementary information [file 41598_2019_55096_MOESM1_ESM.pdf]

Supplementary information for

Evaluation of the dual mTOR / PI3K inhibitors Gedatolisib (PF-05212384) and PF-04691502 against ovarian cancer xenograft models

Simon P Langdon[1*], Charlene Kay[1], In Hwa Um[3], Michael Dodds[1], Morwenna Muir[2], Grant Sellar[4], Julie Kan[5], Charlie Gourley[2], David J Harrison[3]

[1]Cancer Research UK Edinburgh Centre and Edinburgh Pathology, Institute of Genetics and Molecular Medicine, University of Edinburgh, Edinburgh EH4 2XU, United Kingdom.

[2]Nicola Murray Centre for Ovarian Cancer Research, Cancer Research UK Edinburgh Centre, Institute of Genetics and Molecular Medicine, University of Edinburgh, Edinburgh EH4 2XU, United Kingdom

[3]Pathology, School of Medicine, University of St. Andrews, North Haugh, St. Andrews, Fife KY16 9TF, United Kingdom

[4]Wyeth Translational Medicine Research Consortium, Sir James Black Centre, Dow Street, Dundee DD1 5EH, United Kingdom

[5]Pfizer Translational Pharmacology, Oncology, San Diego, USA

[*]Corresponding author: simon.langdon@ed.ac.uk

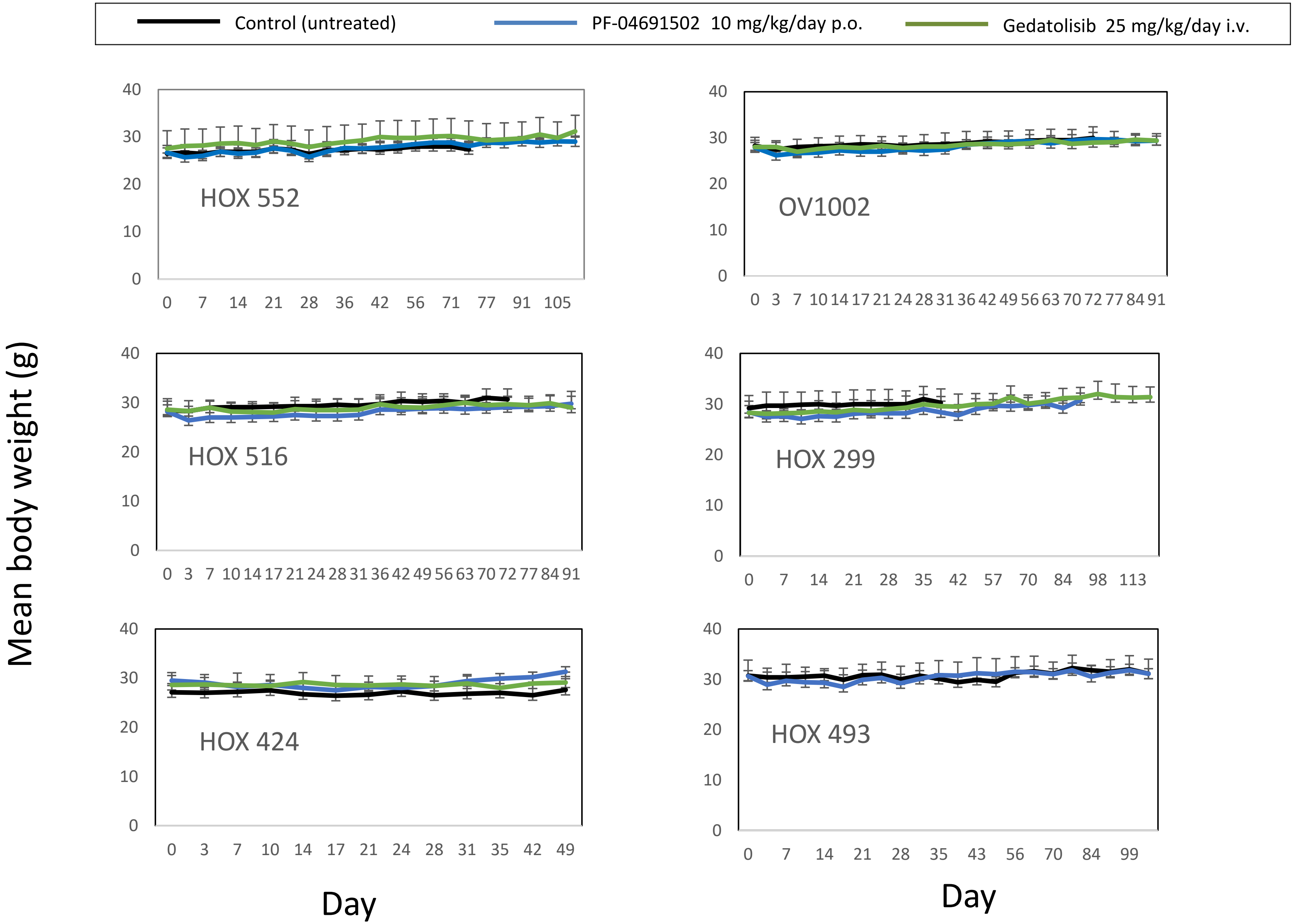

Supplementary Figure 1. Mean body weights (+/- S.D.) for the antitumour studies

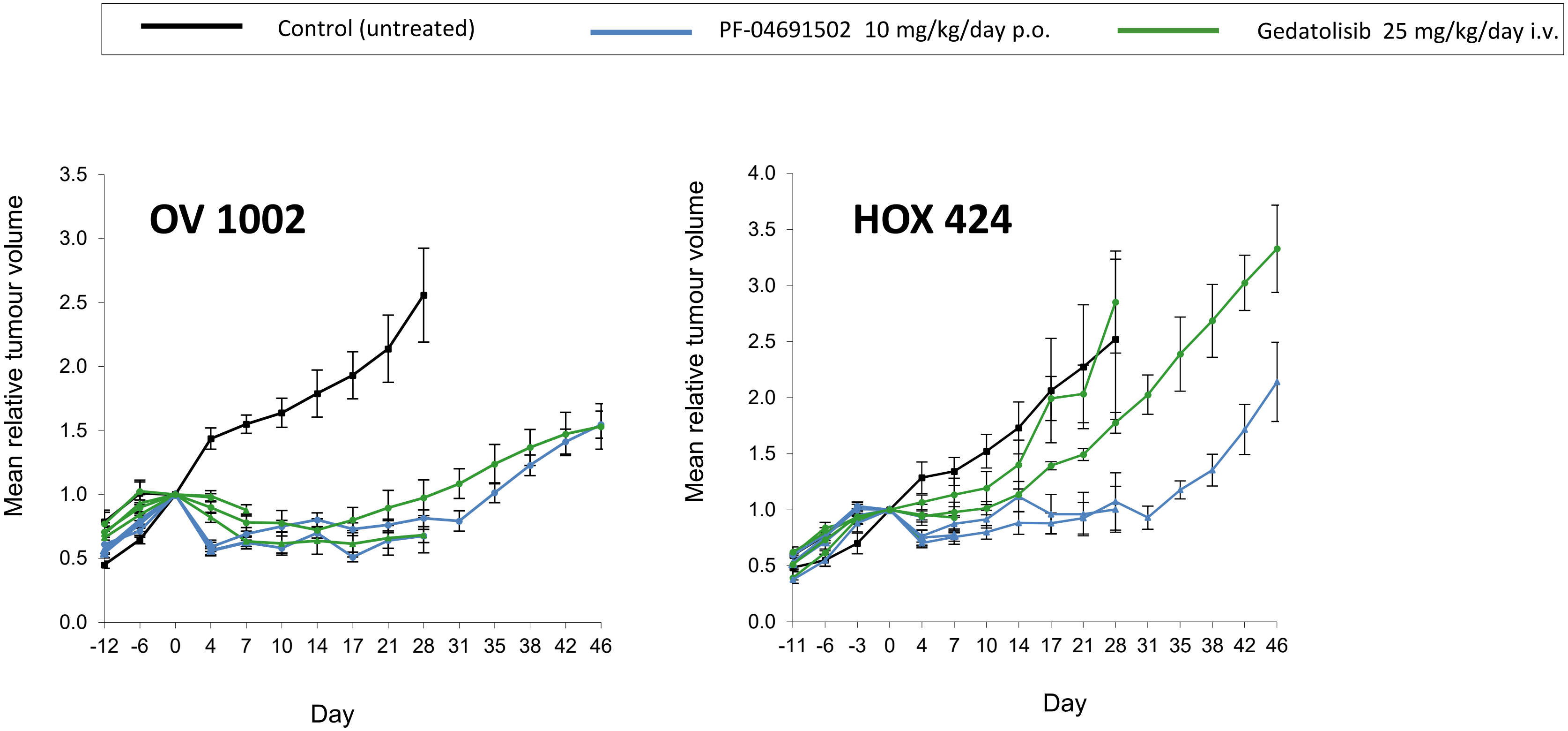

Supplementary Figure 2. Mean relative tumour volumes for the samples collected in the time course expression analysis.

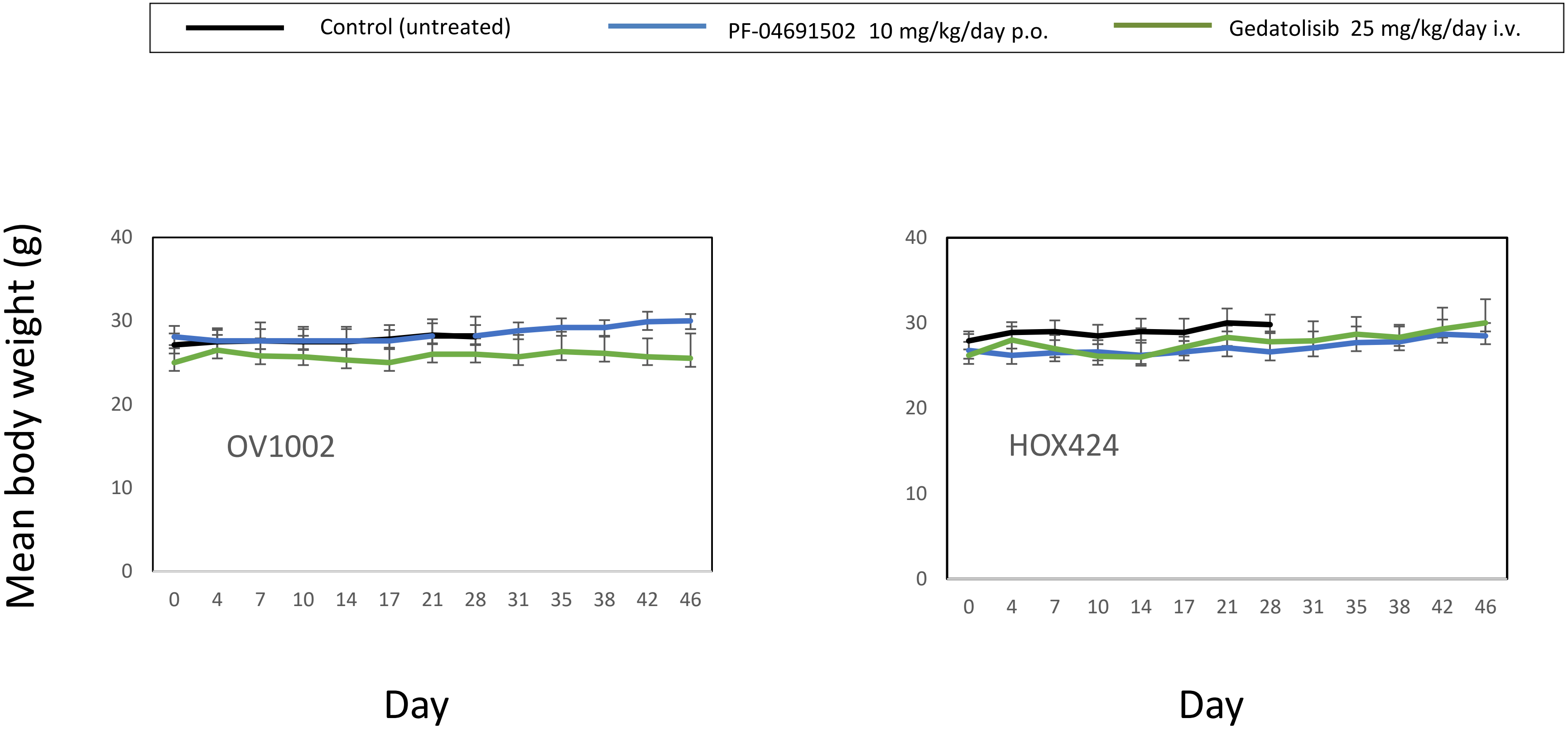

Supplementary Figure 3. Mean body weights (+/- S.D.) for the animals used in the tumour collection study

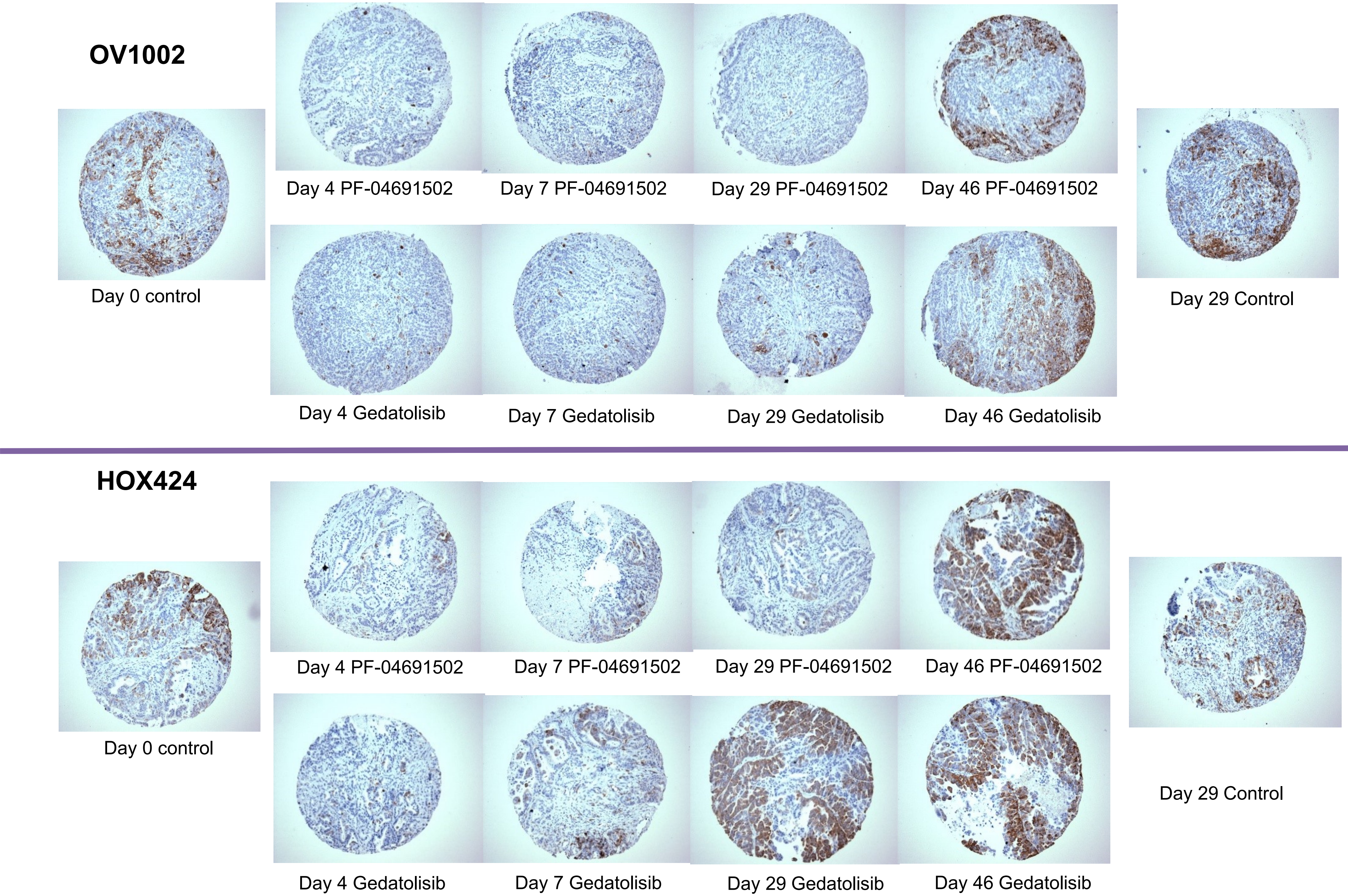

Supplementary Figure 4. Representative images of pS6 (Ser235/236) immunohistochemical staining in TMA cores at each time point. TMA cores were stained as described in Materials and Methods.

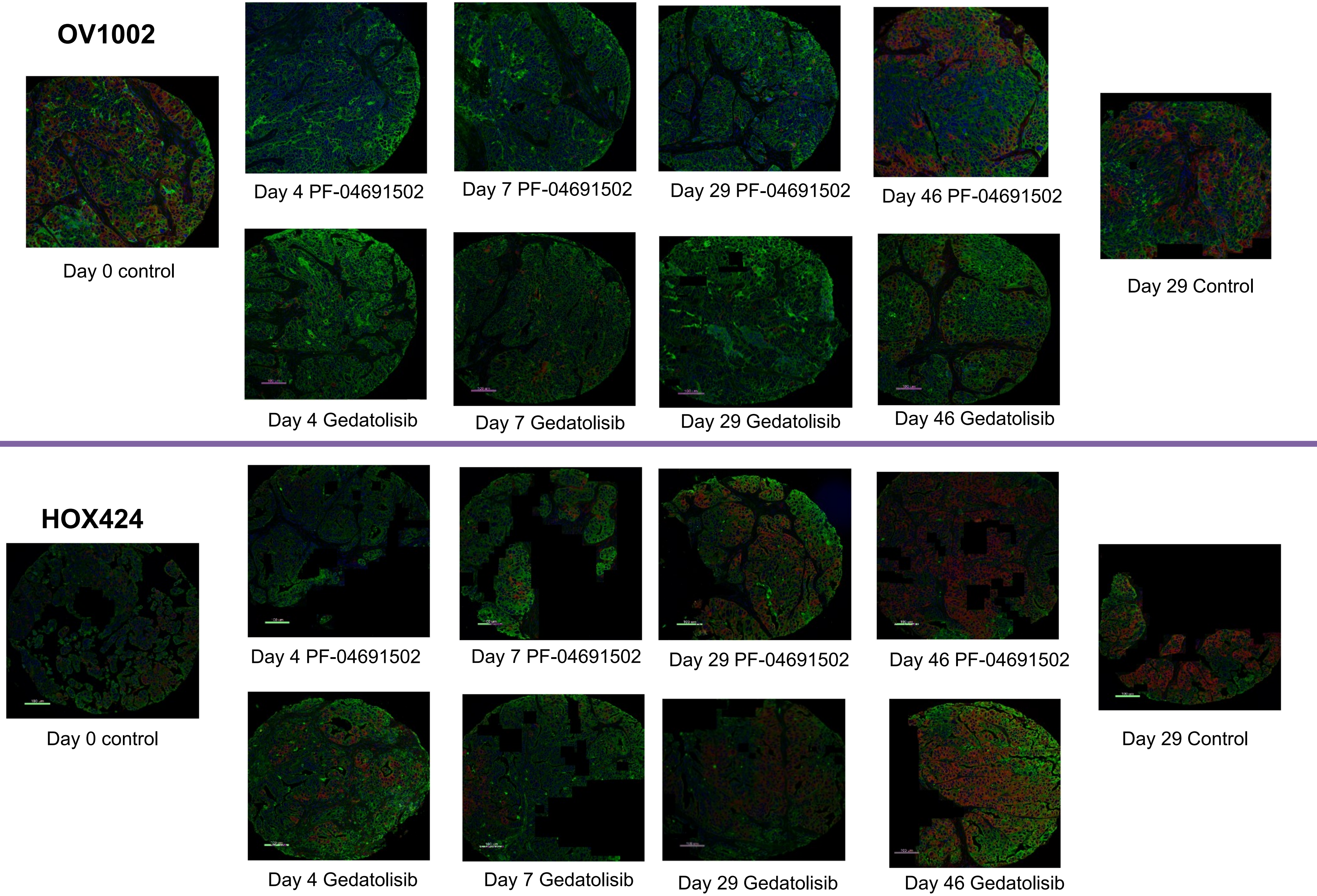

Supplementary Figure 5. Representative images of pS6 (Ser235/236) immunofluorescence staining in TMA cores at each time point. TMA cores were stained as described in Materials and Methods.

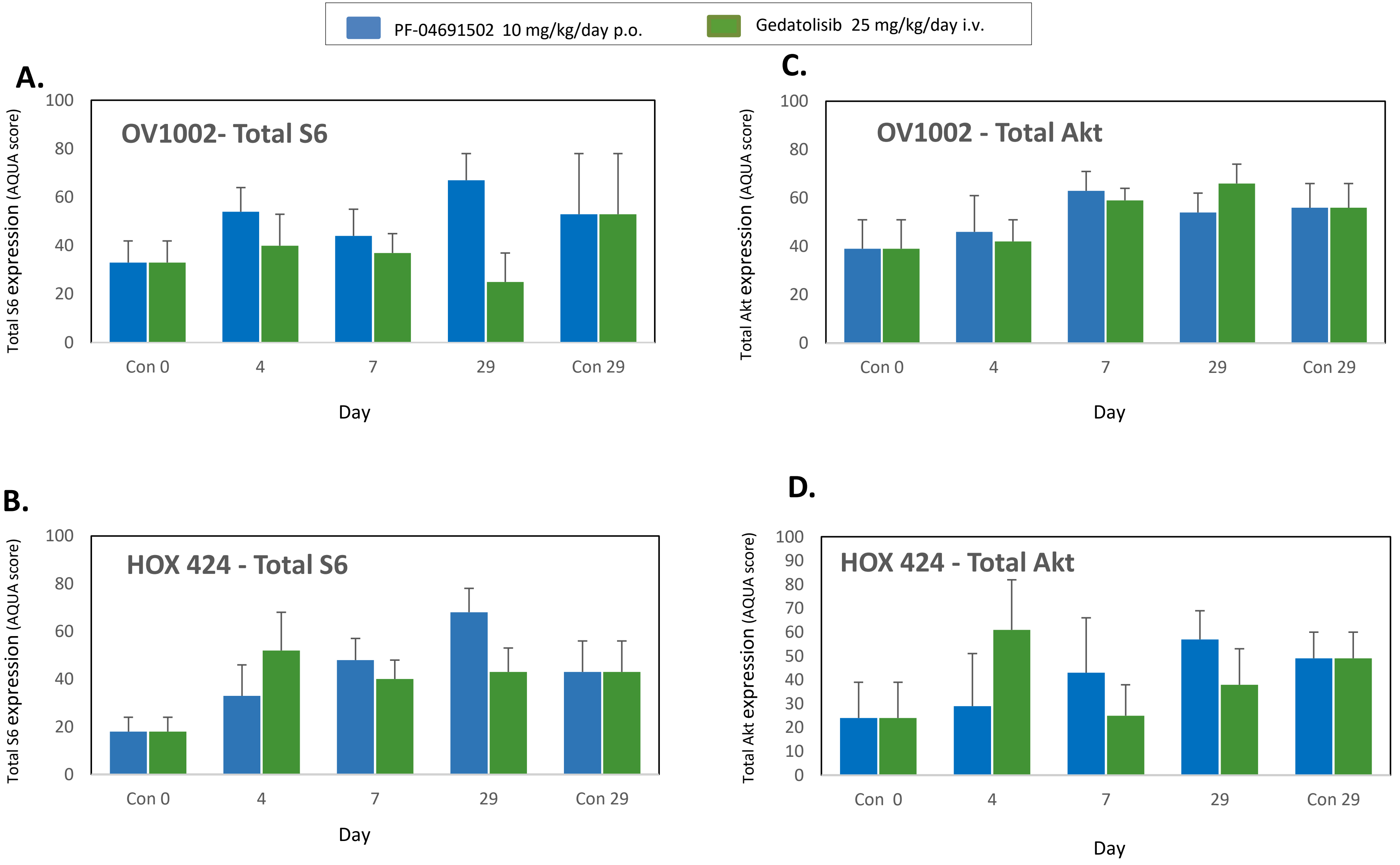

Supplementary Figure 6 Expression of total S6 and Akt in OV1002 and HOV424 xenografts after treatment with PF-04691502 and gedatolisib. A and B: Total S6 expression detected by immunofluorescence in OV1002 (A) and HOX 424 (B) xenograft sections. C and D: Total Akt expression detected by immunofluorescence in OV1002 (C) and HOX 424 (D) xenograft sections. Normalised AQUA scores are shown. Values shown are mean values +/- S.D. for each group of xenografts

Xenograft	pS6/Total S6 ratio	Mean treated tumour volume / mean control volume (Day 7)	Mean control Tumour doubling time (days)	Mean PF-04691502 Treated tumour regrowth rate on cessation of treatment (days)	Ratio of treated / control tumour doubling times
HOX 552	0.26	0.93	38	>79	> 2.1
OV 1002	0.5	0.57	28	21	0.75
HOX 493	0.28	0.76	42	42	1
HOX 424	0.65	0.67	19	10	0.53
HOX 299	0.3	0.68	29	24	1.27
HOX 516	0.2	0.77	21	29	1.4

Supplementary Table 1. Data used to test associations between pS6/total S6 expression and initial tumour response on Day 7 and also regrowth rate on cessation of treatment with PF-04691502. Associations are illustrated in Figures 5C and 5D. Doubling times were calculated by linear regression analysis using data shown in Figure 1. Control doubling time was taken from Day 0, while post-treatment doubling time was assessed from Day 28.
